# Kinetic modelling of serum S100b after traumatic brain injury

**DOI:** 10.1186/s12883-016-0614-3

**Published:** 2016-06-17

**Authors:** A. Ercole, E. P. Thelin, A. Holst, B. M. Bellander, D. W. Nelson

**Affiliations:** Division of Anaesthesia, University of Cambridge, Cambridge, UK; Section for Neurosurgery, Department of Clinical Neuroscience, Karolinska Institutet, Karolinska University Hospital Solna, Stockholm, Sweden; School of Computer Science and Communication, KTH Royal Institute of Technology, Stockholm, Sweden; Section for Anesthesiology and Intensive Care, Department of Physiology and Pharmacology, Karolinska Institutet, Stockholm, Sweden

**Keywords:** S100b protein, Human, Traumatic brain injury, Biomarkers, Kinetics

## Abstract

**Background:**

An understanding of the kinetics of a biomarker is essential to its interpretation. Despite this, little kinetic modelling of blood biomarkers can be found in the literature. S100b is an astrocyte related marker of brain injury used primarily in traumatic brain injury (TBI). Serum levels are expected to be the net result of a multi-compartmental process. The optimal sample times for TBI prognostication, and to follow injury development, are unclear. The purpose of this study was to develop a kinetic model to characterise the temporal course of serum S100b concentration after primary traumatic brain injury.

**Methods:**

Data of serial serum S100b samples from 154 traumatic brain injury patients in a neurointensive care unit were retrospectively analysed, including only patients without secondary peaks of this biomarker. Additionally, extra-cranial S100b can confound samples earlier than 12 h after trauma and were therefore excluded. A hierarchical, Bayesian gamma variate kinetic model was constructed and the parameters estimated by Markov chain Monte Carlo sampling.

**Results:**

We demonstrated that S100b concentration changes dramatically over timescales that are clinically important for early prognostication with a peak at 27.2 h (95 % credible interval [25.6, 28.8]). Baseline S100b levels was found to be 0.11 μg/L (95 % credible interval [0.10, 0.12]).

**Conclusions:**

Even small differences in injury to sample time may lead to marked changes in S100b during the first days after injury. This must be taken into account in interpretation. The model offers a way to predict the peak and trajectory of S100b from 12 h post trauma in TBI patients, and to identify deviations from this, possibly indicating a secondary event. Kinetic modelling, providing an equation for the peak and projection, may offer a way to reduce the ambiguity in interpretation of, in time, randomly sampled acute biomarkers and may be generally applicable to biomarkers with, in time, well defined hits.

## Background

Traumatic brain injury (TBI) is a major health problem worldwide. Data on the true total cost of TBI in Europe are incomplete but even excluding non-hospitalized patients are estimated to be around €33 billion [1]. The brain is uniquely vulnerable to insult due to its high metabolic rate and limited intrinsic energetic reserve, and failure to detect secondary injuries early and act accordingly may contribute to death or serious disability. This has serious socioeconomic implications: For individuals with severe TBI, disability and lost productivity costs outweighing medical and rehabilitation costs by a factor of 4 [2].

Severe primary injuries may evolve and be compounded by subsequent secondary insults and a main objective of neurointensive care unit (NICU) treatment is to predict, detect and prevent further injury. It is therefore of importance to be able to quantify and monitor the effects of both primary and secondary insults over time. Biomarkers of injury are of increasing clinical interest and a number of relatively brain specific molecules have been studied as potential markers of TBI severity [3].

One of the most studied serum biomarkers is S100b, a member of the S100 family proteins which have a wide range of regulatory functions (see review by [4]). S100b is a small oligomeric cytoplasmic calcium binding protein found predominantly in astrocytes. It has been implicated in several cellular processes, particularly those of calcium homeostasis and signal transduction. Levels in the cerebrospinal fluid (CSF) after injury are typically 10–100 times higher than those found in serum [5]. Release into the serum after brain injury could theoretically be the result of several factors including impaired blood brain barrier integrity [6], CSF recirculation to blood via venous drainage [7] and circulation through the recently identified glymphatic system [8]. The rate and temporal profile of release may possibly also be related to the injury type [8, 9]. S100b elimination from serum is thought to be mostly renal [10] with the serum half-life to be in the vicinity of 30–90 min [11, 12].

Unfortunately, other sources of S100b including skeletal muscle, chondrocytes and adipocytes have also been identified [13]. S100b is elevated after extracranial trauma [14] and has also been suggested as marker of malignant melanoma [15]. The extent of extracranial confounding in polytrauma patients with TBI has been discussed [16, 17], but studies suggest that extracranial S100b is quickly eliminated [18, 19]. As a result serum levels after 12 h are most related to TBI outcome [20]. Notwithstanding these caveats S100b has been repeatedly related to outcome in TBI studies [21] although this finding has been inconsistent [22, 23].

If S100b levels change rapidly after injury, then measured concentrations will be very sensitive to sample timing. We postulate that much of the diversity in S100b findings may be due to a limited understanding of its extended release kinetics after TBI, and that the non-standardized sample timing relative to the initial trauma may contribute to diverging interpretations. If this is correct then modelling the kinetics of S100b may significantly increase our understanding of S100b as a biomarker. This study aims to better understand the temporal changes of serum S100b levels after traumatic brain injury by modelling the kinetics using a gamma variate wash-in/out curve- the most plausible function given the above understanding of S100b dynamics to date.

## Methods

This study was approved by the Stockholm County local ethics committee (2009/1668-31/2). No consent was required for this retrospective study. Data from 388 patients ≥15 years of age treated for TBI at the Karolinska University Hospital NICU were extracted from electronic hospital records between 1^st^ January 2005 and 31^st^ December 2009. Patients were treated with standard NICU care as previously reported [20]. The patients included in this study, in part, overlap two previously published papers on S100b from this institute [20, 24].

Serum S100b was routinely sampled upon ICU admission and approximately every 12 h during the ICU stay and the time of blood sampling was carefully recorded. The time of trauma was extracted from the Karolinska TBI database, a prospectively collected and curated database where timings are determined from emergency call and automated ambulance call times.

Two immunoassay methods were used during the study period due to a change in analysis provided at the Dept. of Clinical Chemistry laboratory. Samples through Sept. 2008 were analysed with LIASON-mat S100 system (Diasorin, Sangtec, Italy) and thereafter with Elecsys S100b (Roche Diagnostics, Penzberg Germany). The Elecsys method may exhibit somewhat lower levels, compared to the LIASON-mat, especially at high contractions of S100b [25, 26]. However, good congruence between these methods has also been seen [27, 28] as did the overlap period at the Clinical Chemistry laboratory, Karolinska University Hospital (unpublished results, internal validation report).

Only data 12 h post injury was included in our analysis, as we have previously shown that levels prior to this are poorly related to outcome probably due to contributions from extracranial trauma [20]. Additionally, patients with obvious evidence of secondary injury defined as an S100b increase after peak of >0.05 μg/L/12 h and outliers with uncharacteristic curves were excluded (7 patients). Furthermore, patients were required to have at least 3 samples of S100b and starting within 48 h of trauma. 154 of the original 388 admitted patients during this period remained for the final statistical analysis.

### Statistical analysis

We chose to model the time dependence of the S100b concentration for the *i*-th patient (*S*_*i*_) as a function of time (*t*) as a gamma variate function.

1 $$ {S}_i(t)\kern0.5em =\kern0.5em {A}_i{t}^a exp\left(\hbox{-} t/\beta \right)\kern0.5em +\kern0.5em B $$

This is a hierarchical model where *A*_*i*_ represents a patient-specific amplitude related to the severity of their individual insult and *B* is a baseline physiological S100b level for the population as a whole. The gamma variate is the most simple stochastic model describing a wash-in/out curve [29] and has a characteristic peak-tail shape. The population kinetic parameters *α* and *β* control the shape of the curve. It can be shown that *α* may be interpreted as depending on the number of theoretical “mixing chambers” or turbulence in the mixing process and *β* is related to the ratio of mixing chamber volume to flow [30]. In the limit of no mixing chambers (i.e. an elimination process only), *α* becomes 0 and thus Equation 1 reduces to the form of a decaying exponential with rate constant 1/*β* as expected. It can be shown mathematically that the peak concentration of S100b occurs at time *t*_*peak*_ = *αβ* in this model.

The two parameters *α* and *β* are antagonistic and fitting of data to the gamma variate function using traditional computer techniques is well known to be extremely difficult due to numerical instability, which limits convergence. Because of this and because estimates of the posterior distributions of the parameters and peak time/credible intervals are of clinical interest, we developed a novel nested Bayesian approach.

Markov chain Monte Carlo (MCMC) simulations were carried out using JAGS (version 3.1.0) [31] sampler and rjags package within the R statistical programming language [32]. We used non-informative Gaussian priors for *A*_*i*_, *α* and *β*. We chose non-informative gamma function distributions as priors, *B* and for our estimation of *t*_*peak*_. We demonstrated stability after a 1,000 round burn-in and estimated parameters using three chains over 20,000 iterations each.

## Results

### Patient characteristics

**Fig. 1:**
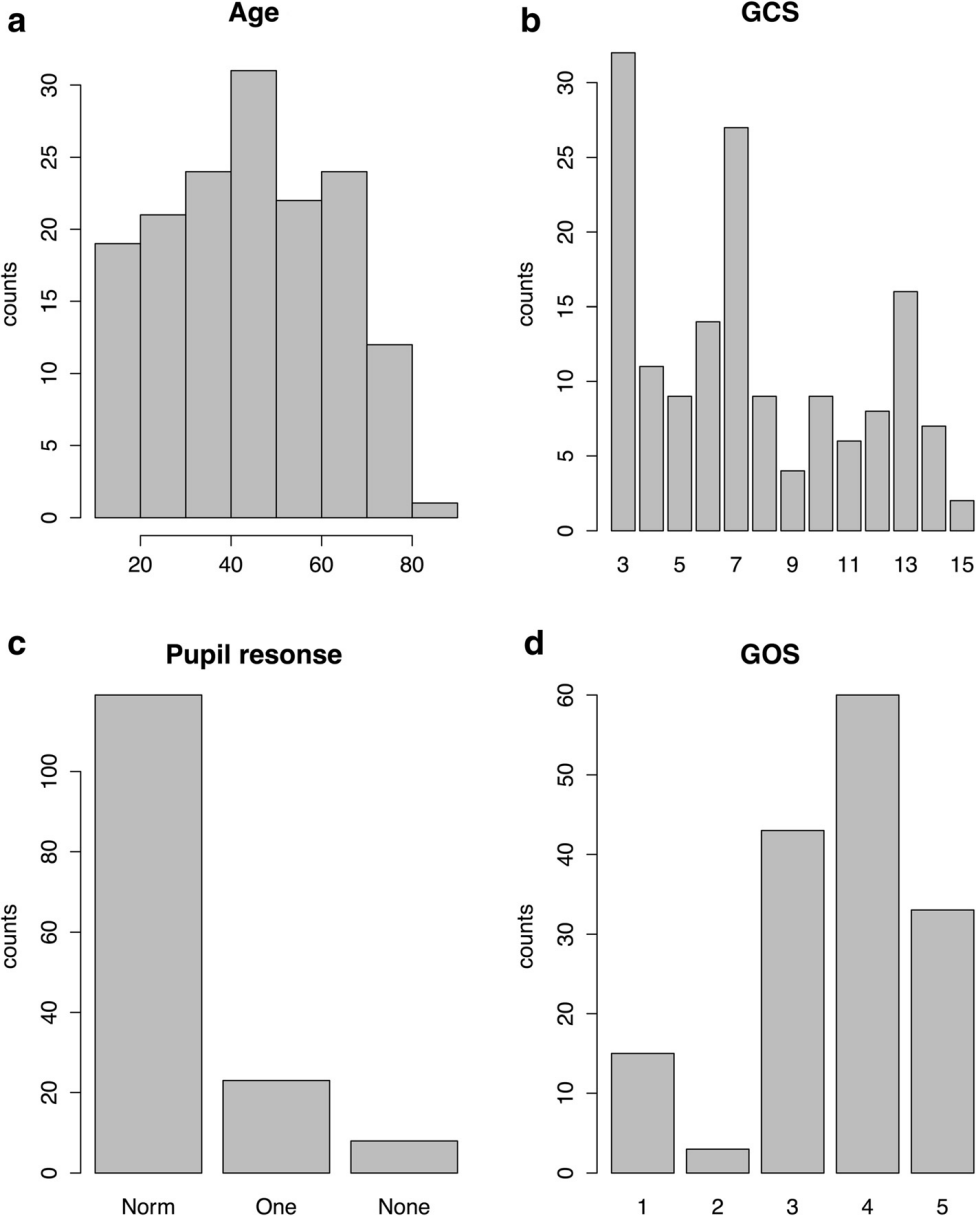
Demographics. **a** Age (**b**) Glasgow Coma Scale (GCS) at admission (**c**) Pupil responses: Normal, One responsive, Non-responsive (**d**) Glasgow Outcome Scale (GOS) barplot.(GOS 1 = dead - GOS 5 = full recovery)

See Table 1 for demographics. The 154 included patients had a mean age of 45 years (range 15–84) and 81 % were males. The median Glasgow Coma Scale (GCS) was 7. Histogram/barplots of age, admission Glasgow Coma Scale (GCS), pupil responses and Glasgow Outcome Scales (GOS) are presented in Fig. 1a-d showing a 9.7 % mortality and 60 % favourable outcome (GOS > 3).

**Table 1:** Demographics

Gender male	81 %
Age (mean)	45
Admission GCS (median)	7
Pupil unilaterally responsive	15 %
Pupils unresponsive	5.3 %
Major Multi-trauma (ATLS criterion)	47 %
Epidural hematoma	23 %
Subdural hematoma	66 %
Traumatic subarachnoid haemorrhage	67 %
Contusion	67 %
Bleeding progression between CT scans	56 %

### Kinetics of S100b

From our MCMC simulations, we obtained posterior estimates for the mean values of *α* = 0.69 (s.d. 0.042; 95 % credible interval [0.61, 0.77]) and *β* = 1.65 day^−1^ (s.d. 0.057; 95 % credible interval [1.54, 1.76]). The mean fitted time to peak was *t*_*peak*_ = 27.2 h (s.d. 0.82; 95 % credible interval [25.6, 28.8]).

The mean background S100b level was estimated to be 0.11 μg/L (s.d. 0.0053; 95 % credible interval [0.10, 0.12]).

**Fig. 2:**
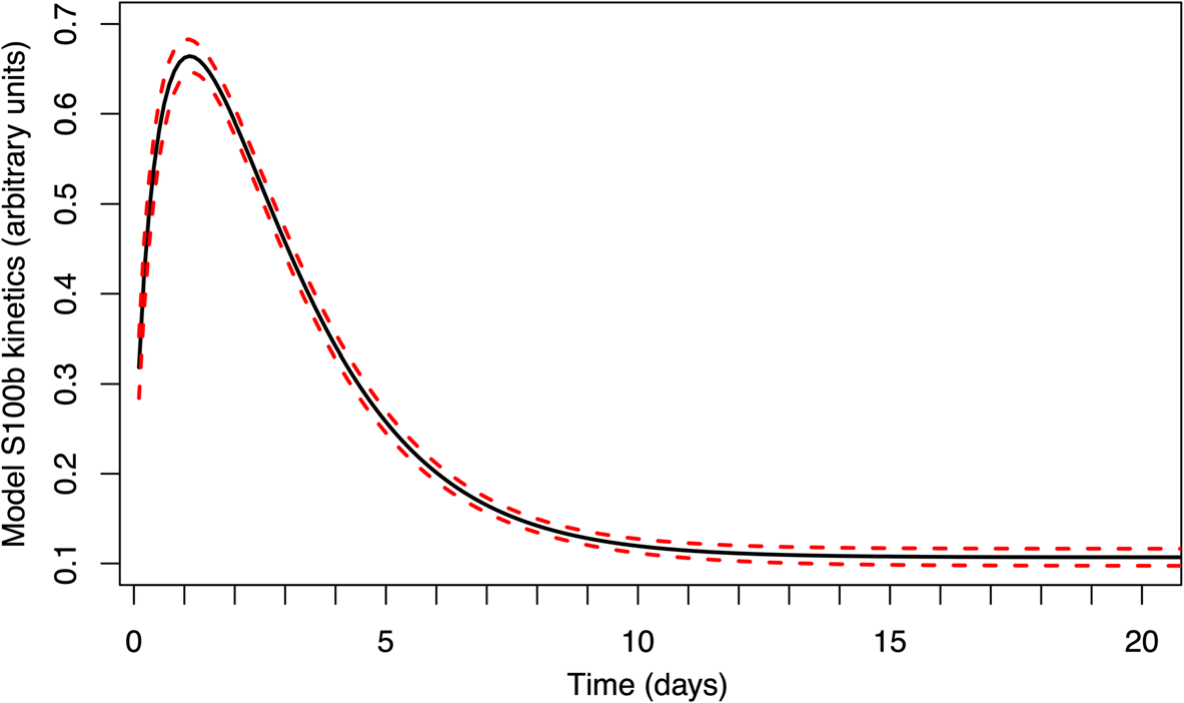
Fitted kinetic gamma variate model. Calculated mean of S100b posterior prediction distribution as a function of time from our model. The dotted lines represent 95 % prediction intervals and within our model are interpreted as the parameter uncertainties excluding the variation from individual patients

Figure 2 shows the posterior prediction for S100b as a function of time with 95 % prediction intervals. This takes into account the variability in the fitted values of *α*, *β* and *B* constrained by the functional form of equation 1. Subject to this constraint, the estimated parameters produce a very tight fit for the average kinetic behaviour.

**Fig. 3:**
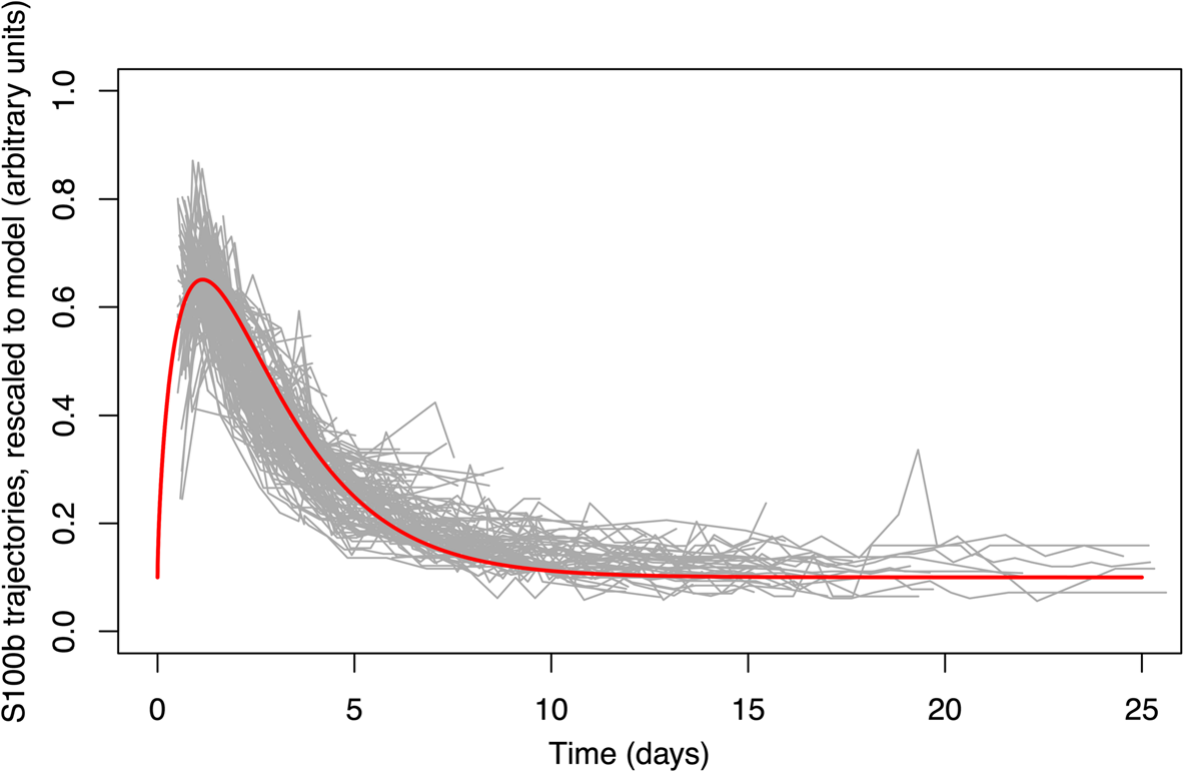
Plot of S100b trajectories for all patients. Re-scaled and translated to minimise mean-square deviation from the from the model curve. The model curve is overlaid

Figure 3 shows the predicted model with normalised individual patient trajectories and overlaid gamma model. From this plot it can be seen that the data generally fits the functional form of Equation 1 well although there is additional inter-patient variability. As the data is rescaled, by patient, some patients will appear to have secondary peaks that in absolute numbers do not exceed the exclusion criteria of >0.05 μg/L.

## Discussion

We have demonstrated that S100b varies strongly with time in the first few days after trauma, with a peak just after day 1. From Fig. 2 it is clear that even excluding individual variability, the rapidly changing concentrations predicted by the kinetics mean that even relatively small differences in sampling time lead to very different measurements, particularly in the first days after injury. This has clear implications for clinical practice and research studies using S100b.

For measurements to be comparable, the timing of early S100b measurements after injury should ideally be standardised- this would be difficult to achieve in clinical practice. Our model offers a method to correct for differences in sampling time. Furthermore, an approach such as ours could find novel application in the detection of subsequent secondary injuries from serial S100b measurements. Given any S100b level >12 h post-injury, deviation of subsequent measurements from the standard kinetic curve over time would be an indicator that a new injury had taken place. Conversely, levels of S100b following the standard curve are reassuring; potentially justifying relaxation of therapeutic targets under circumstances where this may be causing systemic harm, thus individualising treatments.

From our results, we suggest that kinetic studies of biomarkers are essential before evaluation and that some basic principles should be adhered to. Sampling a biomarker too infrequently leads to misinterpretation. In our study, the half time of S100b in serum, which is known to be short, does not explain the values after the initial trauma peak and they must be seen as the net of a slow release process, and possible production, from the injured site in a multi-compartment model. The information content of a biomarker will be related to these changes over time [33]. To ascertain this, initial temporal profiling of a biomarker should be oversampled at a frequency exceeding the characteristic timescale by 2–3 times by analogy with Nyquist-Shannon limit from signal theory [34].

To our knowledge, this is the first study that has attempted to quantify the kinetics of a clinical biomarker in terms of a kinetic model. Additionally, as far as we are aware, this is the first time that such a wash-in/wash-out model which is known to be mathematically problematic has been fitted to observational data using a Bayesian/MCMC approach to yield estimates of the posterior parameter distributions. Such an approach enables the S100b level to be evaluated for any sampling time after a discrete insult such as occurs in trauma. Such a methodology may find applications not just to TBI, but also in the interpretation of S100b after other acute brain syndromes and perhaps in other acute biomarkers outside neuroscience.

This work is necessarily based on an analysis of observational data leading to some limitations. In particular, since there is no accepted gold standard for assessing secondary injury it is therefore likely that some of the patients did experience secondary insults, which will distort our estimates of the kinetic parameters. Whilst we cannot quantify this effect we would not expect this to have a great effect on our estimate of the time to peak. Furthermore, the presence of secondary injuries in our data would principally be expected to contribute to a slower decay and therefore our estimate in some sense represents an ‘upper bound’ to S100b kinetics. In other words, whilst we cannot entirely exclude secondary injuries in a hypothetical patient following the same trajectory as our model, a patient with S100b decreasing less quickly than in our model (or increasing) would be very suggestive of on-going damage i.e. such a temporal behaviour would be highly specific for secondary injury.

It is plausible that the prognostic potential of biomarkers such as S100b would be improved by correcting for the exact timing of the sample using knowledge of the kinetics such as from our study. Alternatively, it seems reasonable that the initial insult of the *j-*th patient would be better described by an estimate of *A*_*j*_ (i.e. insult is related to multiples of the standard value) or the area under the expected curve or the true peak height (both calculable from the model). Our current dataset is too small to confirm this; we are currently collecting a dataset, which we hope will allow us to validate this.

Other studies [35, 36] have observed S100b to follow a simple exponential decay function. However, these works have included very early S100b values, which we argue may in fact be of extracranial origin and should therefore be excluded. Furthermore, most previous work has aggregated early S100b data too coarsely in time to be able to clearly resolve the peak around 27 h. We speculate that S100b from extracranial origin would have an early and fast release to plasma and therefore mimic a first order kinetic with exponential decay. Thus two concurrent processes may be exhibited that need be separated. For the slow release process from brain, we have assumed a more sophisticated kinetic model based on the gamma variate. This has the physical interpretation of assuming that S100b kinetics obeys a wash-in/out process with multiple theoretical compartments in series and would therefore seem physically justified. As we have stated previously, simple exponential elimination is a special case of our model with *α* = 0. However, since our 95 % credible interval for this parameter do not include zero, this provides strong evidence for wash-in/out behaviour. More complex models are of course conceivable. However we do not have data to motivate the choice of any particular alternative and less parsimonious model.

Figure 3 shows that there is considerable variability in the true patient data in addition to that explained by variability the model parameters (Fig. 2). Several factors that could affect the accuracy of the model therefore deserve further consideration. First, we have not evaluated the influence of injury type on the kinetics properties. Hermann et al. [9] have suggested that the time of peak may vary with CT findings. It is probable that S100b release after an epidural hematoma has a different time profile than a contusion or, for example, early typical ICP related art. cerebri posterior infarction. The lack of an unambiguous way of classifying injury type suggests that a very large dataset would be required to address this question specifically. Additionally, the sampling frequency of S100b of around 12 h intervals chosen at our institution is arbitrary and it is possible that a higher sampling rate would help to better model S100b kinetics. However, by chance this is found to be 2.25 the peak time and would therefore seem adequate, if slightly on the low side. Finally, the modelling of the offset value does not extend beyond the NICU period of acute damage. The baseline found in our study of 0.11 μg/L (s.d. 0.0053 μg/L; 95 % credible interval [0.010, 0.12]), is close to the normal reference values of the analysis method (Roche <0.10 μg/L) suggesting a limited effect of the primary injury on S100b levels after 10–15 days.

As stated previously, two different laboratory assays were used to measure S100b during the study period. If of significance, this would be expected to broaden the limits of confidence of the model, which are tight. We are therefore fairly confident that this is not a major limitation. Another factor that could broaden these intervals is uncertain trauma origin times. Given that these are given from ambulance alert times there may be some variation in the timing of trauma to alert time and this would cause a more uncertain peak time. Again, given the tight intervals of the model this would not appear to be a serious limitation.

In addition to S100b, a large number of other brain injury biomarkers including neurofilament (NF), glial fibrillary acidic protein (GFAP), ubiquitin carboxyl terminal hydrolase-L1 (UCH-L1), spectrin breakdown products, cleaved tau (C-tau), neuron specific enolase (NSE) are known and seem to have subtly different pathobiological sensitivities [3], although all respond in some gross way to brain injury. It therefore seems likely that the greatest diagnostic potential will come from studying panels of different markers, enabling clinicians to exploit differences between them to better characterise injury processes. However, since these differences seem to be subtle, a fuller characterisation of the kinetics of these other biomarkers in different disease states is likely to be important too.

## Conclusions

We have modelled the serum kinetics of S100b demonstrated that it changes rapidly in the first days after injury. The interpretation of the biomarker level in relation to the primary injury will therefore be highly influenced by the time from trauma and even small differences in timings may lead to inaccurate assessment of the magnitude of the injury. This has been little explored in the biomarker literature and may greatly affect the results and possibly interpretations of other studies. We have demonstrated that S100b concentrations vary rapidly in the first 24–48 h after injury indicating that an optimal and standardized window for sampling must be identified. Alternatively, if variably timed measurements of S100b are to be used for prognostication or research it is crucial to take the timing into account by comparison with a reference curve such as the one presented in this work. Furthermore, monitoring for systematic deviations above the predicted curve in serial measurements may offer a clinical method for detecting the occurrence of secondary injuries. Further validation against outcome is required. However, we believe that kinetic considerations are of general importance when using biomarkers with rapidly changing levels over timescales of clinical interest.

## Abbreviations

CSF, cerebrospinal fluid; GCS, Glasgow Coma Scale; GOS, Glasgow Outcome Scale; MCMC, Markov chain Monte Carlo; NICU, neurointensive care unit; TBI, traumatic brain injury
